# Renal Function in Children on Long Term Home Parenteral Nutrition

**DOI:** 10.3389/fped.2019.00137

**Published:** 2019-04-16

**Authors:** Assylzhan Messova, Robert Dziubak, Jutta Köglmeier

**Affiliations:** Unit of Nutrition and Intestinal Failure Rehabilitation, Department of Paediatric Gastroenterology, Great Ormond Street Hospital for Children, London, United Kingdom

**Keywords:** paediatric intestinal failure, long term parenteral nutrition, renal function, glomerular filtration rate, Schwartz formula

## Abstract

**Objectives:** To assess renal function in pediatric intestinal failure (IF) patients on long term home parenteral nutrition (HPN).

**Methods:** Children who received HPN for a minimum of 3 years between 2007 and 2017 were identified from the IF clinic of a large tertiary referral center. Estimated glomerular filtration rate (eGFR) was calculated using the Schwartz formula at discharge on HPN, after 6 months, 1, 2, and 3 years.

**Results:** Twenty five patients (40% male) fulfilled the inclusion criteria. The indications for HPN were due to an underlying motility disorder in 56% (14/25), enteropathy in 24% (6/25), and short bowel syndrome in 20% (5/25). At the start of HPN 80% (20/25) had a normal eGFR. Four children (17%) had an abnormal eGFR. In the group of patients with normal eGFR at the start of HPN 30% (6/20) had at least one episode of decreased eGFR in the following 3 years, however there was no significant decline in eGFR at the end of the 3 year study period. Overall there was no statistically significant deterioration of eGFR in the study population (*p* = 0.7898).

**Conclusion:** In our cohort of children on long term HPN no significant decline of eGFR could be demonstrated within 3 years of starting PN.

## Introduction

Children with prolonged or irreversible intestinal failure (IF) require long term parenteral nutrition (PN) (1). Good quality of life can be achieved on home PN (2). PN is usually infused overnight by a skilled carer. Depending on the underlying etiology of IF many children are able to eat and drink or tolerate at least enteral nutrition given via a feeding device. However, a group of young patients is fully PN dependent. Although lifesaving long term PN is by far no panacea and associated with well-known complications such as central venous catheter (CVC) related blood stream infections, loss of central venous access, liver disease, pulmonary embolism, metabolic disturbances and bone disease (3–5).

In recent years, strategies have been developed to protect the liver as much as possible.

Modern lipid solutions such as SMOF or Omegaven have contributed to a significant reduction in IF associated liver disease (IFALD) (6–8).

Carbohydrate concentrations are kept to the minimum required and PN is prescribed in a cyclical manner as early as possible allowing for several hours off intravenous substrate infusion during the day.

However, children who are unable to absorb enteral fluids are at risk of dehydration during the PN break, particularly those with large fluid and electrolyte requirements such as patients with ultra-short bowel syndrome.

Chronic dehydration could have a negative impact on renal function (9). The formation of calcium oxalate stones in the kidneys is a common complication in adults and children with short bowel syndrome (10). However, evidence from the literature on adults suggests that long-term PN in general, irrespective of the underlying etiology of IF, is associated with serious renal impairment (9–13).

Moukarzel et al. were amongst the first to report on renal dysfunction in children (14). The authors noted a decrease in glomerular filtration rate (GFR), but were unable to establish an underlying mechanism. Since then only a few papers have been published referring to pediatric patients who developed a decline in renal function on treatment with prolonged PN (15–17).

The aim of our study was to investigate the effects of long term home PN on the renal function in our cohort of children with prolonged or irreversible IF.

## Methods

This was a cross-sectional, retrospective cohort study. Data were collected over a 3 year period. Patients were recruited from the IF rehabilitation clinic of a large tertiary pediatric referral center in the United Kingdom. Children who had been on home PN for a minimum of three consecutive years between 2007 and 2017 were included into the study. Based on the underlying etiology of their IF patients were grouped into three categories: short bowel syndrome, enteropathy, and motility disorder.

Patient demographics including age, gender, weight and height and the duration of home PN, body mass index (BMI), and serum creatinine levels were obtained from the medical records.

The dietetic notes were consulted to establish if children were fully PN dependent or received additional calories from eating food or liquid enteral feeds. The children received variable nutritional support according to tolerance dependent on their underlying pathology. Some received a simple mineral and vitamin supplement taken by mouth alongside oral solid food whilst others drank a specialized calorie supplement. Liquid enteral feeds were either taken orally or given via a gastric or jejunal artificial feeding device.

Parenteral energy and protein were prescribed according to the 2005 ESPGHAN guidelines (18).

Weight and standing height were routinely measured at every clinic visit in keeping with the hospital clinical practice guidelines.

Standard equipment used were medical electronic scales and a stadiometer for length/height.

The GFR was calculated using the Schwartz formula: Schwartz Formula: eGFR = 36.5 × height (length) divided by serum creatinine and expressed as milliliters per minute per 1.73 m^2^ (19). Renal function was defined as normal if the eGFR was >90 mL/min/1.73 m^2^. Decreased renal function was classified as mild with an eGFR ranging from 89 to 60 mL/min/1.73 m^2^, moderate when the eGFR was 59–30 mL/min/1.73 m^2^, and severe when the eGFR was <30 mL/min/1.73 m^2^.

All children were regularly followed up in the outpatient clinic. Patients were included into the study after informed consent had been obtained. The study was approved by the hospital research and development department as an audit. Data were collected retrospectively from the case notes. We calculated the children's eGFR at the start of home PN, at 6 months, 1, 2, and 3 years of intravenous nutrition at home. We documented which patients had an abnormal or normal eGFR at the start of home parenteral nutrition (HPN) and the end of the audit—at 3 years of being on HPN. We looked separately at the group of which children experienced at least one decrease of eGFR during first 3 years of HPN and established if they had an eGFR within the normal range or outside at the start of HPN.

We compared the rates of decrease in eGFR between groups of children who were born prematurely, received total or partial HPN, who had preexisting kidney disease, the underlying IF diagnosis and those who received medication with a potential effect on renal function such as corticosteroids or immunosuppressive agents at the start and end of the study period, as well as during the 3 year interval for those patients with a decreased eGFR at the start of HPN.

We also compared the rates of decrease in eGFR of patients who had their CVC locked with Taurolock with those who did not, as reduced CVC related blood stream infections in the group of patients on Taurolock could play a potential role in preservation of renal function.

Data were analyzed with R version 3.2.3 (R Core Team, 2015, Vienna). Categorical variables are presented as frequencies and continuous data as medians. We used the chi-square test to compare categorical variables (**Table 3**). We compared continuous variables using the Wilcoxon signed-rank test (**Table 4**) and Kruskal-Wallis test to check if there was a correlation between the change of eGFR at 6 months, 1, 2, and 3 years and the underlying diagnosis, and differences between BMI and underlying diagnosis at 6 months, 1, 2, and 3 years of HPN. All tests were two-sided and the *p*-value was set to 0.05.

## Results

Twenty five children (40% male) fulfilled the inclusion criteria and were enrolled into the study. The median age at the start of home PN was 30 months (range 10–54 months). Fourteen patients (56%) had an underlying motility disorder, six (24%) were diagnosed with an enteropathy and five (20%) suffered from short bowel syndrome (Table 1).

**Table 1:** Patient demographics and underlying IF etiology, diagnosis and content of parenteral nutrition, medication prescribed.

Patient number	25
Male to female ratio	10/25 (40%)
Age at start of PN, months	30
**Diagnosis**	
Short bowel syndrome	5/25 (20%)
- Malrotation with volvulus	1/25
- Gastroschisis	1/25
- Necrotising enterocolitis	3/25
Enteropathy	6/25 (24%)
- Tufting enteropathy	4/25
- Microvillus inclusion disease	1/25
- Protein losing enteropathy	1/25
Motility disorder	14/25 (56%)
- Total aganglionosis	1/25
- Chronic intestinal pseudo obstruction	5/25
- Neuropathic dysmotility	8/25
Total parenteral nutrition	12/25 (48%)
Partial parenteral nutrition	13/25 (52%)
Corticosteroids	8/25 (32%)
Immunossuppressant	10/25 (40%)
Taurolock	7/25 (28%)

Just under half of the children (12/25; 48%) received total PN including full calorie and maintenance fluid volume, the remainder tolerated some oral and/or enteral feeds (13/25; 52%) and received partial PN.

Six patients (24%) had an underlying renal pathology at the start of HPN including antenatally diagnosed hydronephrosis, bilateral renal dysplasia, renal tubulopathy, recurrent urinary tract infections, and nephrocalcinosis.

Apart from the patient with nephrocalcinosis, who suffered from tufting enteropathy, all other children with preexisting renal disease had motility disorders. Out of the 25 children, 8 (32%) were prescribed corticosteroids in the study period. Ten patients (40%) received immunosuppressant therapy; one for tufting enteropathy, one with protein losing enteropathy, two children with chronic intestinal pseudoobstruction, and 6 with neuropathic dysmotility. We also documented when patients had been started on Taurolock line locks, which was the case in 7 (28%) (Table 1).

Eight children (32%) had an abnormal creatinine at the start of HPN and four (17%) a decreased eGFR (Table 2).

**Table 2:** Annual GFR results.

**Renal function degree (mL/min/1.73m^**2**^)**	**Start**	**6 month**	**1 year**	**2 year**	**3 year**
eGFR >90	20/24 (83%)	18/23 (78%)	19/24 (79%)	18/24 (75%)	22/25 (88%)
eGFR 89–60	4/24 (17%)	5/23 (22%)	3/24 (13%)	6/24 (25%)	2/25 (8%)
eGFR 59–30	–	–	2/24 (8 %)	–	1/25 (4%)

The eGFR was decreased at 6 months, 1, 2, and 3 years for 5 (22%), 3 (13%), 6 (25%), and 2 (8%) of the patients, respectively, but there was no significant difference between those points in time. As we collected data retrospectively eGFR could not be calculated in rare circumstances as one patient was wheel chair dependent and height was hence not adequately recorded.

In the group of 20 children who started HPN with a normal GFR, 6 (30%) had at least one episode of a decreased eGFR during the first 3 years on HPN. Of the children who experienced a reduction in eGFR during the study period, patients with an underlying enteropathy or motility disorder had a 1 or 2 fold reduction in eGFR compared to those with short bowel syndrome, who had a more pronounced decrease of eGFR ranging from a 2 to 4 fold reduction. Children with short bowel syndrome are more prone to dehydration due to higher fluid requirements which is the likely explanation for this (17, 20).

The median BMI at the start of HPN, at 6 months, 1, 2, and 3 years was 16.40, 17.20, 16.45, 16.55, and 16.00, respectively. The median change of eGFR compared to the baseline eGFR at the start of HPN at 6 months, year 1, 2, and 3 was 3.3%, 2.1%, 0.40%, and 9.2%.

When comparing the different underlying etiology of those patients with IF who started the study with a normal eGFR and experienced at least one episode of a decreased eGFR during the study period, 23% of the children who had a motility disorder had eGFR decreased within 3 years, 25% of children with enteropathy had a decrease, and 67% of patients with short bowel syndrome had decreased GFR at least one time during 3 years.

We examined other factors that could affect renal function including preexisting renal disease, full or partial HPN and any medication prescribed. In our cohort of patients, 16.7% with renal pathology but normal eGFR at the start of HPN had at least one episode of decreased eGFR during the 3 year follow up period. However, 35.7% of children without kidney disease also had a decreased eGFR at some point in the study interval. Thus, renal disease for patients on HPN had no significant effect on renal function (Table 3; *p*-values based on Chi-square test).

**Table 3:** Abnormal GFR amongst the different factors in patients who started HPN with a normal GFR.

**Patients who started HPN with normal GFR**			
	**when No**	**when Yes**	***p*****-value**
Prematurity	4/16 25%	2/4 50%	0.7144
Total PN	3/11 27.3%	3/9 33.3%	1
Kidney Disease	5/14 35.7%	1/6 16.7%	0.7494
Steroids	5/14 35.7%	1/6 16.7%	0.7494
Immuno supressors	4/13 30.8%	2/7 28.6%	1
Taurolock	5/15 33.3%	1/5 20%	1

Thirty-three percent of patients on total PN experienced at least one episode of decreased eGFR compared to 27% of children on partial PN. However, this was not significant (*p* = 1).

Half of the children who were born premature had a decreased eGFR. However, when compared to full-term infants there was no statistically significant difference. Prematurity therefore appears not to have a major impact on renal function in children who go on to require PN for a prolonged period of time (Table 3).

We investigated the effect of the use of steroids, immunosuppressant medication and Taurolock central venous catheter locks on renal function. We could again not demonstrate any significant changes in the groups receiving such treatments and in those who did not. Thus, treatment with steroids and immunosuppressants whilst receiving PN appears not to lead to a significant decrease in kidney function (Table 3). There was also no statistically significant difference between groups of children who did or did not receive Taurolock line locks.

We looked at those children who started HPN with a low GFR, as well as those who experienced a decrease in eGFR within the 3 years, and which patients had a normal or abnormal eGFR at the start of HPN and the end of the audit. The type of PN prescribed (partial or total), preexisting renal disease, prematurity, treatment with steroids, immunosupressants, or Taurolock did not result in a statistically significant difference of abnormal eGFR results in either of the groups.

The level of change of eGFR at 6 months, 1, 2, and 3 years of HPN when compared to baseline (at start of HPN) was again not statistically significant. Similarly, we could not find any significant changes in the rate of eGFR change between children born prematurily, receiving total PN, with an underlying kidney disease, those being prescribed medications and different IF diagnosis (Table 4; *p*-values based on Wilcoxon signed-rank test).

**Table 4:** Changes of GFR at 6 months, 1, 2, and 3 years of HPN when compared to baseline.

	**Change GFR 6M**			**Change 1Y**			**Change 2Y**			**Change 3Y**		
	**when No**	**when Yes**	***p*-value**	**No**	**Yes**	***p*-value**	**No**	**Yes**	***p*-value**	**No**	**Yes**	***p*-value**
Prematurity	7.06	−22.99	0.69	0.56	32.26	0.405	3	13.48	0.94	7.32	18.36	0.48
Total PN	−14.56	13.67	0.235	4.54	88.9	0.265	−3.12	4.54	0.37	0.71	12.84	0.24
Kidney disease	10.95	−1.5	0.745	3.83	−7.17	0.820	3.34	−7.84	0.97	9.21	2.1	0.87
Steroids	−7.03	17.38	0.10	−7.29	4.54	0.105	−8.76	3.86	0.19	−15.73	17.3	0.09
Immuno-suppressant	−7.03	7.13	0.283	4.64	−3.46	0.508	−0.18	0.4	1	−0.22	11.75	0.34
Taurolock	10.87	−9.42	0.918	4.07	−7.49	0.493	3	−13.48	1	11.1	−22.45	0.90

Similarly, we didn't find significant differences between BMI and the percentage of change of eGFR at 6 months, 1, 2, and 3 years of HPN and underlying diagnosis (enteropathy, short bowel syndrome and motility disorder), prematurity, gender, total vs. partial PN, kidney disease, and medication (steroids, immunosuppressants, Taurolock).

## Discussion

The aim of this study was to assess renal function in pediatric IF patients on long term PN, a treatment available since the late 1960s (21, 22).

The number of adults and children receiving this life saving therapy has since grown dramatically (12).

Modern IF rehabilitation has achieved to reduce the severity and number of well-known complications and home PN is allowing age appropriate development with acceptable quality of life (3–5).

In particular liver protection has been a major focus achieving dramatic reduction of the number of patients requiring a liver or combined liver and small bowel transplant (8). However, although home PN in childhood is now considered a safe treatment with a high probability of long-term survival, the mortality is increased if not managed by an expert team, in patients younger than 2 years of age and severe underlying IF pathology such as ultra-short bowel syndrome, myopathic pseudoobstruction and congenital mucosal diseases (13). Adequate assessment and monitoring of children prescribed PN by a multidisciplinary nutrition support team is hence recommended to keep the number of complications to a minimum (23).

A decrease of renal function in adult patients with benign IF is known to occur when frequent episodes of dehydration occur (16). Little data on the impact of long-term PN on the kidneys in the pediatric IF patients have been published, and there is little emphasis on renal protective measures mentioned in the current literature.

However, like adults, children are at risk of dehydration during the PN break, particularly in those with large fluid and electrolyte requirements or if enteral absorption is, depending on the underlying IF etiology, significantly compromised.

In cyclical PN urine production occurs predominantly during the overnight infusion and many parents report that their children pass little urine during the day. Chronic dehydration could negatively impact on renal function (9). Formation of renal calcium oxalate stones is seen both in adults and children with short bowel syndrome who have their colon in continuity (10). However, evidence from the literature on adults suggests that long-term PN in general, irrespective of the underlying etiology of IF, is associated with serious renal impairment (11). Studies on preterm infants receiving total PN have shown that renal function as early as 30 days after the start of PN is reduced compared to neonates of the same gestational age who tolerated enteral feeds (24). In adult IF patients the use of nephrotoxic drugs and particularly sepsis appear to play a significant role in the development of renal dysfunction. However, the underlying pathology remains poorly understood in a significant proportion of long term PN patients. In children with IF concerns about the deterioration of renal function were first expressed in the early 1990s (14). The authors noted a decrease in GFR but were unable to establish an underlying mechanism. Only a few smaller pediatric case series have been published since (15, 16).

However, a recent study performed in Finland on 80 patients with pediatric onset IF has shown a significantly increased risk of impaired renal function associated with the duration of PN and length of remaining small bowel in children with short bowel syndrome (17).

We were concerned that our patients experience the same problems and we therefore studied glomerular function in those who received home PN for at least 3 years.

In contrast to the results of the previous authors we could not demonstrate a statistically significant reduction in estimated GFR despite a similar duration of home PN.

The reasons for this may simply be that the patients in our clinic have reassuringly not experienced a decline in renal function during the study period. However, we used the Schwartz formula to estimate the GFR in our children rather than measuring the true GFR as we felt this would have not been practicable in this age group. GFR describes the volume of fluid filtered from the renal glomerular capillaries into the Bowman's capsule per unit time and is equal to the clearance rate when any solute is freely filtered, and is neither reabsorbed nor secreted by the kidneys (25). As GFR cannot be measured directly ideal filtrations markers such as Inulin are used for the measurement of GFR. However, inulin or the use of radioactive tracers require an injection of a labeled substance, several blood and often urine samples, which is difficult to achieve in young children, as they can be uncomfortable and invasive (26).

Alternative markers of GFR such as Cystatin C only requiring a simple blood test, which could be obtained from the CVC at the time of routine blood testing, were not available in our center at the time of the study (20).

Schwartz published a method for estimating GFR from serum creatinine, the patient's height and a proportionality constant and is currently considered the best method for estimating GFR in children (19).

In most of our patients the serum creatinine level was decreased before and after the start of home PN. IF can lead to malnutrition and loss of muscle mass (27, 28). Serum creatinine concentration is dependent on the volume of endogenous creatinine distribution, the rate of production in relation to muscle mass and the amount excreted primarily through glomerular filtration.

In patients with a reduced muscle bulk creatinine values are hence proportionally less for a given level of GFR compared to those with more muscle.

Comparing the cohort of patients from the authors from Finland with our children, we had a proportionally smaller number of patients with short bowel syndrome. Significant reduction in bowel length made their patients more prone to the development of renal dysfunction (17). However, we could not demonstrate a significant difference according to underlying IF pathology in our study. This may be genuine or simply a reflection of the relatively small sample size in each group. It is hence possible that eGFR did not adequately reflect renal function in at least some of our patients.

Our cohort of patients had a higher proportion of patients with motility disorders with a tendency to progress over time, leading to the need for PN later in childhood. The median age at the start of home PN was 30 months. In comparison children develop short bowel syndrome mostly in infancy or early childhood. Young children with immature kidney function may hence be more prone to adverse effects of PN on renal function. In addition CVC related blood stream infections are common in children with short bowel syndrome (29). Sepsis itself has been shown to be associated with decreased kidney function in patients on home PN (30). Potential contributing factors are bacteraemia and the use of nephrotoxic antibiotics used to treat the infection. In individuals with severe oliguria serum creatinine concentration becomes primarily a function of production and volume of distribution within the body (31). It has been shown that the expansion of lean body mass with intravenous nutrition is challenging (27). This may be one of the reasons why our patients had low serum creatinine levels despite receiving adequate nutrition from PN.

Some of our patients required steroid medication to reduce intestinal inflammation. Corticosteroids can cause muscle atrophy and hence contribute to a decrease in serum creatinine levels (32). However, we had patients both with a high and low serum creatinine level whilst receiving steroids and no statistical significant difference in eGFR could be found.

Although we were very encouraged that we could not demonstrate a significant reduction in renal function in our cohort of children with IF, we would like to continue to monitor our young patients for this potentially serious complication of long term PN. In the future we are aiming to estimate GFR by using the Schwartz formula and Cystatin C levels and compare the two.

Cystatin C is protein produced by all nucleated cells in the human body.

It is freely filtered by the glomerulus and reabsorbed and catabolized by the tubular epithelial cells (33). Only a small amount is excreted in the urine. Cystatin C generation is less affected by age and sex compared to serum creatinine. However, urinary Cystatin C clearance cannot be measured and it is difficult to study factors affecting its clearance and generation (31). Higher levels have been associated with a high CRP or BMI, hypothyroidism and steroid use (34, 35). Some evidence in the literature supports that serum levels of Cystatin C are a better marker to estimate GFR. However, it is currently not validated in children (30). When compared with the Schwartz formula is likely to be at least of equal value and height measurements are not required, which can be advantageous in young children who are not able to stand up yet, those with spinal deformities or wheel chair dependent. A prospective study comparing eGFR and Cystatin C will not only allow us to get a better understanding about renal function in children receiving long term PN, but also help to inform the discussion about the best possible method for monitoring this group of challenging patients.

We conclude that in our cohort of children on long term HPN no significant decrease of GFR could be demonstrated within 3 years of starting PN. Further studies in a larger group of patients using Cystatin C as well as eGFR are needed.

## Ethics Statement

The authors confirm that the study was registered with the hospital research and development department (R&D). The hospital R&D department confirmed registration as an audit and therefore external ethical approval did not have to be obtained. The authors confirm further that the project did not infringe on any patient confidentiality or privacy. As data was collected retrospectively from clinic notes and test based on standard practice, no additional burden or risk was placed on the patients. The study did not involve any clinically significant departure from usual clinical care. No data were collected from patients which were sensitive or overly time consuming to the patients. Informed consent for participation was obtained in writing from all participants/their legal carers.

## Author Contributions

AM has made substantial contributions to the data collection from medical records, the analysis and interpretation of data for the work and drafting the first version of the manuscript. RD has made substantial contributions to the conception and design of the work; performance of statistical analysis of collected material and interpretation of data. JK holds the intellectual property of the work and was the scientific supervisor during the entire study period. JK supported and supervised the interpretation of data, literature review, advised on the design of the study and writing up the paper. All patients included in the study attend the intestinal failure rehabilitation clinic of GOSH by JK and written consent to enter their results into the study was taken in the outpatient department by the parent or carer holding parenteral responsibility. JK took part in drafting the work and final approval of the version to be published.

### Conflict of Interest Statement

The authors declare that the research was conducted in the absence of any commercial or financial relationships that could be construed as a potential conflict of interest.
